# Sinapic-Acid-Loaded Nanoparticles Optimized via Experimental Design Methods: Cytotoxic, Antiapoptotoic, Antiproliferative, and Antioxidant Activity

**DOI:** 10.1021/acsomega.4c00216

**Published:** 2024-09-17

**Authors:** Fatma Şayan Poyraz, Gülşah Akbaş, Dilek Duranoğlu, Serap Acar, Banu Mansuroğlu, Melike Ersöz

**Affiliations:** † Department of Molecular Biology and Genetics, Faculty of Art and Sciences, Yildiz Technical University, Istanbul 34349, Turkey; ‡ Department of Chemical Engineering, Faculty of Chemical and Metallurgical Engineering, Yildiz Technical University, Istanbul 34210, Turkey; § Department of Bioengineering, Faculty of Chemical and Metallurgical Engineering, Yildiz Technical University, Istanbul 34220, Turkey; ∥ Department of Molecular Biology and Genetics, Faculty of Arts and Sciences, Demiroglu Bilim University, Istanbul 34394, Turkey

## Abstract

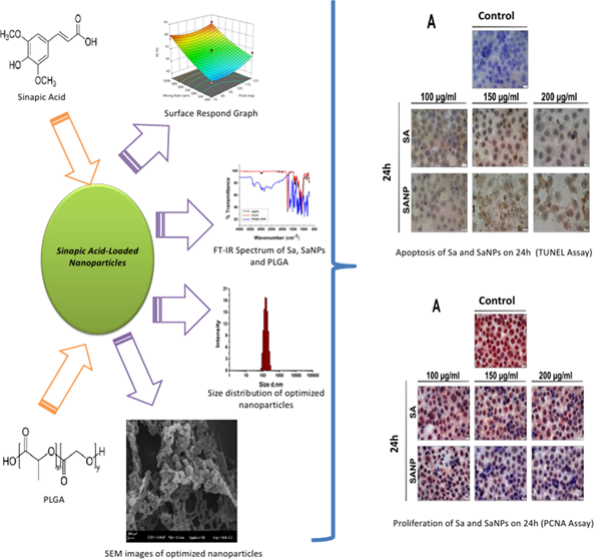

Nanoparticles are frequently investigated as carrier systems that increase the biological activities of hydrophobic molecules, especially by providing them with water solubility. Sinapic acid (Sa), commonly found in plants, is a phenolic compound with a wide spectrum of biological activities and extensive pharmacological properties. The aim of this study was the synthesis/characterization of optimized sinapic-acid-loaded poly(lactic-*co*-glycolic acid) (PLGA) nanoparticles (SaNPs) to improve the solubility of sinapic acid (Sa) that limit its use in the biological system and investigate the biological activities of these nanoparticles in the breast cancer cell line. For this purpose, sinapic-acid-loaded PLGA nanoparticles were obtained and optimized by experimental design methods. Then, cytotoxic (MTT method), antiapoptotic (terminal deoxynucleotidyl transferase dUTP nick end labeling (TUNEL) assay), antiproliferative (immunocytochemically by PCNA assay), and antioxidant activities (superoxide dismutase (SOD) and catalase activities, glutathione, malondialdehyde (MDA), and caspase-3 levels) of optimized nanoparticles were examined comperatively with free drug on MCF-7 cells. The IC_50_ values of the SaNPs (170.6 ± 3.6 nm size) in MCF-7 cells were determined at 180, 168, and 145 μg/mL for 24, 48, and 72 h, respectively, and at these doses, the nanoparticles did not show any toxic effect on the MCF10A cell line. Treatment of Sa and SaNPs at doses of 24 and 48 h showed a statistically significant reduction in the PCNA level in MCF-7 cells, with an increase in the number of cells leading to apoptosis. In MCF-7 cells treated with SaNP at concentrations of 150 and 200 μg/mL for 24 h, MDA levels were significantly increased, SOD activities were significantly decreased, and reduced glutathione (GSH) and catalase levels were increased compared with control groups. The findings of this study indicate that polyphenolic compounds can contribute to the design of drugs for treatment by forming nanoparticle formulations. The developed nanoparticle formulation is thought to be a useful model for other hydrophobic biological active substances.

## 1 Introduction

Sinapic acid, a minor hydroxycinnamic acid derivative found in plants, is a phenolic chemical and part of the phenylpropanoid family.^1^ Various in vitro/in vivo studies showed that sinapic acid has anti-inflammatory,^2^ antimicrobial,^3^ cardioprotective,^4^ antihyperglycemic,^5^ anticancer,^6^ anxiolytic,^7^ hepatoprotective,^8^ neuroprotective,^9^ and antioxidant activities.^10^ Despite the promising properties of sinapic acid, its use is limited due to the hydrophobic nature of the molecule.^1^ On the other hand, aqueous formulation of sinapic acid (Sa) can be prepared as nanoformulations that provide improved bioavailability, controlled drug release, and dispersion of drugs in aqueous environments while protecting the molecule from degradation conditions.^11^ These nanoformulations, which are capable of controlling the long-term release of the sinapic acid molecule and the stability in the physiological environment, have great potential in improving the distribution of the active substance more efficiently. Among the polymers used in the preparation of this nanoformulation, a poly(lactic-*co*-glycolic acid) (PLGA) copolymer has high biocompatibility, exhibits inert properties in the physiological environment, and does not show toxicity in biological systems.^12^

In addition, PLGA, which is tried in FDA- and EMA-approved clinical studies, is widely used as an active substance carrier, and nanoparticles produced from this polymer can even pass through capillaries, thanks to their nanometer size.^13^ While cancer-related mortality ranks top globally, breast cancer is one of the most frequent types of cancer. Today, different traditional methods are used for subtypes of breast cancer, such as hormone therapy, chemotherapy, radiation, and surgery. Therapy options are often decided based on tumor subtype and the cancer stage. However, due to drug resistance and inadequate absorption of anticancer medications, these procedures produce poor efficacy and significant side effects. As a result, other therapeutic techniques are required to overcome the limitations of standard approaches, such as polymeric nanoparticles, photodynamic therapy, photothermal therapy, and so on.^14,15^ Polymeric nanoparticles, one of the alternative methods, have been extensively studied in cell cultures, particularly as anticancer drug delivery systems.

The process of entrapment of drug molecules into nanoparticles is influenced by a variety of process parameters; thus, it is critical to first identify the most effective parameters, then examine the effect of selected parameters, and finally determine the optimum preparation conditions systematically for developing effective drug-loaded nanoparticles. For the first time, we studied the influence of process parameters on the production of sinapic-acid-loaded PLGA nanoparticles using two-step experimental design methodologies. The Packett–Burman experimental screening design method was applied first in order to determine the most effective parameters in sinapic acid entrapment efficiency and nanoparticle size, and then the Box–Behnken experimental design, one of the response surface methods, was constructed with the three main process parameters obtained from screening design; hence, effects of each parameter and their interactions were investigated comprehensively.

Consequently, the optimum process conditions to obtain the smallest particle size and the highest entrapment efficiency were determined. The biocompatibility of the optimized nanoparticles in the MCF10A nontumorigenic breast cell line and the cytotoxic effect on the MCF-7 breast cancer cell line were also examined. As a result of the optimum doses determined, the antiproliferative effect was determined by proliferating cell nuclear antigen (PCNA) assay, and the apoptotic effect was determined by the transferase-mediated dUTP end labeling (TUNEL) method in the same cell lines by using these doses. Among biochemical parameters, superoxide dismutase (SOD), malondialdehyde (MDA), reduced glutathione (GSH), caspase-3, and catalase activities were investigated.

## 2 Materials and Methods

### 2.1 Materials

Sigma-Aldrich (St. Louis) provided PLGA (lactide/glycolide = 50:50, inherent viscosity 0.45–0.60 dL/g, *M*_w_ ∼24–38 kDa), poly(vinyl alcohol), sinapic acid, dichloromethane, and ethanol. All of the chemicals and solvents used in the nanoparticle synthesis were analytical grade and did not require additional purification. MCF-7 human breast cancer and MCF-10A nontumorigenic human breast cell lines were acquired from the American Type Culture Collection (ATCC, Manassas, VA).

### 2.2 Methods

#### 2.2.1 Synthesis of Sinapic-Acid-Loaded PLGA Nanoparticles

Sinapic-acid-loaded PLGA nanoparticles were prepared by a single emulsion-solvent evaporation technique according to the literature.^16^ First, 80 mg of sinapic acid was dissolved in a 1:1 (v/v) dichloromethane (DCM) and ethanol mixture. The PLGA was dissolved in 0.5 mL of DCM. Sinapic acid and PLGA solutions were combined and added to poly(vinyl alcohol) (PVA) solutions at various flow rate 1 ratios (Table 1). Sonication was carried out for the emulsification of the aqueous phase with the organic phase (1:1 (v/v) dichloromethane (DCM) and ethanol mixture). After the sonication, the emulsified solution was added to a 0.1% PVA solution using a peristaltic pump with different flow rate 2 ratios (Table 1). Hence, nanoparticles were obtained from the solution. Having evaporated the solvent by stirring with different mixing rates for 4 h at room temperature, the nanosuspensions were centrifuged at 10,000 rpm for 40 min at 4 °C (Beckman Coulter Allegra X-30R Centrifuge, Germany). The supernatant was collected, and the nanoparticles were rinsed with 35 mL of distilled water 3 times. The recovered supernatants were employed in an indirect assessment to estimate the entrapment efficiency. Sinapic-acid-loaded nanoparticles (SaNPs) were freeze-dried at 0.01 mbar, – 70 °C for 48 h, without any cryoprotectant. The lyophilized nanoparticles were kept at −80 °C until usage.

**Table 1:** Factors and Levels Used in the Plackett–Burman Design

			levels	
factors		low (−1)	center point (0)	high (+1)
*X*_p1_	amount of PLGA (mg)	75	100	125
*X*_p2_	concentration of PVA (%)	1	1.5	2
*X*_p3_	sonication power (W)	60	80	100
*X*_p4_	sonication time (min)	30	60	90
*X*_p5_	flow rate 1 (mL/min)	4	5	6
*X*_p6_	flow rate 2 (mL/min)	1	2	3
*X*_p7_	mixing rate (rpm)	400	800	1200

#### 2.2.2 Plackett–Burman Screening Design

The Plackett–Burman (PB) screening design was implemented in order to examine a large number of process parameters in a minimum number of experimental runs.^17^ By this way, we could determine the factors that require further investigation. Design Expert software (V. 13.0, Stat-Ease Inc., Minneapolis, MN) was used to develop the regression model. The linear equation of the model is given as follows
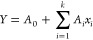
1where *Y* is the predicted response, *A*_0_ is the intercept term, *A_i_* is the linear effect, *x_i_* is the value of independent variables, and *k* is the number of the independent variables.

Synthesis parameters like the amount of PLGA (*X*_p1_), PVA concentration (*X*_p2_), sonication power (*X*_p3_), sonication time (*X*_p4_), flow rate 1 (flow rate of PLGA-SA mixture into 1.5% PVA, *X*_p5_), flow rate 2 (flow rate of emulsified solution into 0.1% PVA, *X*_p6_), and the mixing rate (mixing rate during the solvent evaporating, *X*_p7_) were examined as factors (independent variables), while entrapment efficiency (*Y*_1_) and particle size (*Y*_2_) were selected as response (dependent variables) for Plackett–Burman design. The level of seven independent variables used for Plackett–Burman Design is shown in Table 1.

#### 2.2.3 Box–Behnken Design

Following the determination of the most effective parameters using Plackett–Burman design, a 3-factor 3-level Box–Behnken (BB) experimental design technique was used to investigate the effects and interactions of the selected factors. The quantity of PLGA (*X*_b1_), mixing rate (*X*_b2_), and sonication power (*X*_b3_) were chosen as the Box–Behnken design parameters. Table 2 shows the variables, responses, and levels utilized in the Box–Behnken design.

**Table 2:** Factors and Levels Used in the Box–Behnken Design

			levels	
factors		low (−1)	center point (0)	high (+1)
*X*_b1_	amount of PLGA (mg)	75	100	125
*X*_b2_	mixing rate (rpm)	400	700	1000
*X*_b3_	sonication power (W)	60	80	100

Design Expert software (V. 13.0, Stat-Ease Inc., Minneapolis, MN) was used for the development of the regression model. Experimental data were fitted to the quadratic model given below
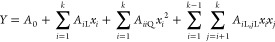
2where *Y* is the estimated response, *A*_0_ is the intercept term, *A*_*i*L_ is the linear effect, *A*_*ii*Q_ is the quadratic effect, and *A*_*i*L,*j*L_ is the binary linear interactions. Furthermore, *x_i_* and *x_j_* are the value of factors and k is the number of factors.^17^

#### 2.2.4 Characterization of Polymeric Nanoparticles

##### 2.2.4.1 Entrapment Efficiency (EE) and Drug Loading (DL)

The entrapment efficiency and drug loading were measured indirectly by spectrophotometric analysis of the supernatant produced after centrifugation.^16^ The quantity of unentrapped sinapic acid in the supernatant solution was evaluated using UV–vis spectroscopy at 306 nm, and the concentration of sinapic acid was determined using a previously developed standard calibration curve. The entrapment efficiency and drug loading were calculated using the formulae below.
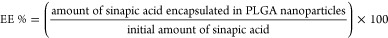
3
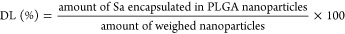
4

##### 2.2.4.2 Mean Particle Size (*Z*-Ave), Zeta-Potential (ζ), Polydispersity Index (PDI), and Surface Morphology

Particle size and ζ-potential values of sinapic-acid-loaded nanoparticles were measured using a Zetasizer (Nano ZS, Malvern Instruments, U.K.) applying dynamic and electrophoretic light scattering methods.^18^ The tests were conducted by using a nanoparticle solution diluted with ultrapure water. Measurements were done in triplicate at 25 °C. Atomic force microscopy (AFM) was used to evaluate the shape and surface morphology of the optimized nanoparticles (SPM 9600, Shimadzu, Japan), X-ray powder diffraction (XRD) (X’Pert PRO Powder Diffractometer, PANalytical, Austria), scanning electron microscopy (SEM) (EVO LS10, Carl-Zeiss, Germany), and transmission electron microscopy (TEM) (JEM 1220, JEOL, Japan). AFM studies were performed in a dynamic mode in two-dimensional (2D) and three-dimensional (3D) for the morphological analysis of nanoparticles. Five microliters of a nanoparticle solution was dropped into a freshly cleaved mica surface and incubated for 5 min. The mica surface was cleaned with distilled water, dried for 20 min, and then analyzed. The crystalline and/or amorphous structure of the SaNPs was evaluated by X-ray powder diffraction (XRD). Powder XRD patterns of samples were analyzed at room temperature, with the XRD spectra recorded between 3 and 60° (2θ). SEM was performed with an acceleration voltage of 10 kV, and images were taken after Au coating with a magnification of 60,000×. TEM was performed with an acceleration voltage of 10 kV, and images were taken at 30,000×. The samples were negatively stained with a phosphotungstic acid solution (2%, w/v) and dried in air at room temperature before the samples were loaded in a microscope.

##### 2.2.4.3 Fourier Transform Infrared (FTIR) Spectroscopy

Fourier transform infrared spectroscopy was used to analyze functional groups on the surface of nanoparticles in the universal attenuation total reflectance (ATR) mode.^19^ The FTIR spectra of free sinapic acid, free PLGA, and SaNPs were obtained with 16 scans per sample and 4 cm^–1^ resolutions ranging from 650 to 4000 cm^–1^.

##### 2.2.4.4 Drug Release Profile in the PBS Solution at pH 7.4 and pH 5.5

The in vitro release of SaNPs was performed at pH 7.4 and pH 5.5 (tumor microenvironment), using a modified version of the previously published dissolving technique.^20^ Initially, 5 mg of lyophilized SaNPs was suspended in 10 mL of phosphate buffer saline at pH 7.4 and pH 5.5 by vortexing until thoroughly dispersed. Samples were collected over a set period of time and then centrifuged at 10,000 rpm and +4 °C for 20 min. Supernatants were examined at 306 nm with a UV-spectrophotometer to determine the amount of Sa per milliliter. After each sampling, the solution was refreshed with 10 mL of a new buffer.

#### 2.2.5 Cell Culture Conditions

A human breast cancer cell line MCF-7 (ATCC, HTB-22) and nontumorigenic breast epithelial cell line MCF-10A (ATCC, CRL-10317) were obtained from American Type Culture Collection (ATCC, Rockville, MD), and grown according to the instructions of ATCC. MCF-7 cells were cultured in Dulbecco’s modified Eagle’s medium (DMEM)/Ham’s Nutrient Mixture F-12 medium (1:1) (Sigma-Aldrich, St. Louis, MO) supplemented with 10% fetal bovine serum (FBS) (Sigma-Aldrich), 100 U/mL penicillin–streptomycin, and 0.2 mM l-glutamine. Cells were maintained at 37 °C in a humidified incubator containing 5% CO_2_. The medium was changed every 2 days during incubation.

MCF-10A cells were cultured in an MEBM Mammary Epithelial Cell Growth Basal Medium Bullet Kit (CC-3151 from Lonza), supplemented with 10% horse serum. Cells were maintained under the same conditions with MCF-7. The medium was changed every 3–4 days during incubation. The morphology of the cells was examined with Zeiss Zen phase contrast microscopy.

##### 2.2.5.1 Cell Viability Assay

Assesment of cell viability was assessed using MTT (3–(4,5-dimethylthiazol-2-yl)-2,5-diphenyltetrazolium bromide) assay.^21,22^ Both cells were seeded into 96-well plates as 1 × 10^4^ cells/mL. Following the incubation, the medium was changed to a fresh medium containing a series of dilutions (0–1000 μg/mL) of Sa and optimized SaNP (containing 0–1000 μg/mL SA). After 24, 48, and 72 h of incubation, the medium was removed from each well, and the MTT reagent was added and incubated at 37 °C for 3 h. The MTT solution was then collected, and formazan crystals generated by the increased mitochondrial dehydrogenase enzyme were dissolved by adding 0.1 mL of DMSO, and the absorbance was measured at 570 nm using a microplate reader. Untreated cells served as control cells. Metabolic activity values are shown as the mean ± SD of at least three measurements. Cell viability (%) was estimated using the following equation
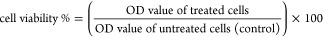
5

##### 2.2.5.2 Measurement of Cell Proliferation

MCF-7 cells were grown on coverslips in 24-well tissue culture plates (1 × 10^4^ cells/mL). After incubation, a series of dilutions of Sa and SaNPs (containing 100, 150, 200 μg/mL Sa) were added to the culture and cultured at 37 °C for 24 and 48 h. After 24 and 48 h, cells were rinsed with phosphate-buffered saline (PBS) and fixed for 5 min at −20 °C with methanol before adding the blocking solution. A proliferating cell nuclear antigen (PCNA) primary antibody (Neomarkers) was administered at 1:150 dilution and incubated overnight at 4 °C. The cells were then rinsed, and then biotinylated secondary antibodies, streptavidin, and biotinylated horseradish peroxidase (Invitrogen) were added. AEC (Invitrogen) kit was used to detect immunoreactivity, hematoxylin–eosin was used to counterstain, and samples were mounted on the slide using mounting media (Lab Vision). The cells were photographed using an Olympus BX-50 bright-field microscope. A minimum of 10 fields was randomly chosen, and marked and unmarked cells were counted in each. The percentage of immunoreactive cells ((the number of immunoreactive cells/total cells) × 100) was expressed.

##### 2.2.5.3 Detection of Apoptosis by TUNEL Assay

Apoptosis was detected according to a terminal deoxynucleotidyl transferase-mediated dUTP end-labeling (TUNEL) assay kit (Millipore). First, MCF-7 cells were seeded on coverslips and were incubated with three different concentrations (containing 100, 150, 200 μg/mL Sa) of the Sa and SaNP for 24 and 48 h. Cells were fixed with methanol, and the equilibration buffer was immediately added to the cells. The TdT enzyme and reaction buffer were mixed, and cells were incubated with this mixture at 37 °C for 1 h. After that, an anti-digoxigenin conjugate was applied to the cells. The reaction was observed by using a diaminobenzidine (DAB) system, and hematoxylin–eosin staining was used as a counterstain. A minimum of 10 fields were randomly selected, and marked and unmarked cells were counted in each field. The percentage of apoptotic cells ((the number of apoptotic cells/total cells) × 100) was expressed.

##### 2.2.5.4 Oxidative Stress and Cellular Antioxidant Activity

All biological systems continually create reactive oxygen species (ROS) during aerobic metabolism. Intracellular antioxidant activity and oxidative stress parameters were determined by assessing the reactive oxygen species (ROS) generation, superoxide dismutase enzyme (SOD) activity, reduced glutathione (GSH) levels, malondialdehyde (MDA) levels, total protein concentration, and catalase activity. In addition, the calorimetric activity of caspase-3, a biochemical parameter that leads to death as a result of oxidative stress, was examined. Cells were seeded in T25 flasks at a concentration of 1 × 10^6^ cells per flask. After cells achieved standard confluency, a fresh medium containing different concentrations (100, 150, 200 μg/mL) of the Sa and SaNPs was added to the culture and incubated for 24 and 48 h. Cells were extracted with trypsin and PBS at 24th and 48th h to produce cell lysates for analysis. The total protein content in lysates was determined by using the bicinchoninic acid technique (Intron BCA Smart Kit) with bovine serum albumin (BSA) as the standard.

##### 2.2.5.5 Determination of Malondialdehyde (MDA) Activity

The thiobarbituric acid reactive substances (TBARS) test, which detects malondialdehyde (MDA), is one of the most often used techniques for detecting ROS formation within cells.^23^ In the TBARS assay, the MDA-TBA products are formed by the reaction of MDA and TBA under high temperatures (90–100 °C) and acidic conditions. To begin, 100 μL of cell lysates was combined with 0.2 mL of a TBA reactive solution, which included 0.375% (w/v) TBA and 0.15% (w/v) trichloracetic acid (TCA). The mixture was then heated for 15 min and centrifuged. Following centrifugation, the reaction mixtures were colorimetrically analyzed at 535 nm with a microplate reader. The results were presented as nanomoles of MDA/mg protein in cells.

##### 2.2.5.6 Determination of Superoxide Dismutase (SOD) Activity

The intracellular SOD enzyme activity technique uses xanthine and xanthine oxidase to produce superoxide radicals. Nitro blue tetrazolium (NBT) transforms into a blue formazan, which serves as an indication for the measured reaction.^24^ To produce the reaction mixture; xanthine, nitro blue tetrazolium, sodium carbonate, BSA, EDTA, and XO enzyme was combined. A hundred microliters of cell lysates was added to 490 μL of the reaction mix and incubated at 25 °C for 20 min. The reaction was halted by adding 0.2 mL of 0.8 mmol CuCl_2_ to the liquid. The absorbance of formazan was measured at 560 nm using a blank containing all of the reagents except the cell lysate. SOD activity was reported as units per milligram of protein.

##### 2.2.5.7 Determination of Reduced Glutathione (GSH) Activity

Boyne and Ellman described a colorimetric technique for measuring total glutathione (GSH) and oxidized glutathione (GSSG) activity.^25^ Metaphosphoric acid was added to 200 μL of the cell lysate for deproteinization. To reduce glutathione (GSH), 1 mmol 5,5-dithiobis(2-nitrobenzoic acid) was diluted in a 1% sodium citrate solution (Ellman reactive) and applied to cell lysates. The absorbance was measured at 412 nm.

##### 2.2.5.8 Caspase-3 Activity

The caspase-3 colorimetric method is based on the hydrolysis of the acetyl-Asp-Glu-Val-Asp peptide substrate by caspase-3, resulting in the separation of *p*-nitroaniline (Ac-DEVD-pNA). *p*-Nitroaniline gives a high absorbance at 405 nm. As a result, the released *p*-nitroaniline concentration is in parallel with the abs at 405 nm.^26^ In the study, caspase-3 levels of cell lysates were measured using the colorimetric caspase-3 assay kit (Abcam ab39401).

##### 2.2.5.9 Catalase Activity

Catalases produce molecular oxygen as a byproduct of the oxidation of hydrogen peroxide. The activity of the enzyme is dependent on the structural conformation of the three basic domains. A three-dimensional structure is regulated due to a reduced NADPH bound to the moiety of the enzyme in the active site, the NADPH binding domain, and a complex secondary structure formed by interlocking long peptide rings during tetramerization. The activity of the catalase enzyme is determined by observing the changes in absorbance resulting from the consumption of H_2_O_2_ at pH 7.0 and 25 °C.^27^

#### 2.2.6 Statistical Analyses

Data were provided as means ± standard deviation (SD) from at least three different experiments performed in triplicate. The statistical analysis was carried out using GraphPad Prism version 6 (GraphPad Software, La Jolla, CA). The one-way ANOVA test was utilized to determine significant differences in cell proliferation and apoptosis studies. The IC_50_ values were also determined with GraphPad Prism. The ANOVA test was used to compare statistical differences in oxidative stress and cellular antioxidant activity assessments between the control and treatment groups, with *p*-values less than 0.05 indicating statistical significance.

## 3 Results and Discussion

### 3.1 Plackett–Burman Screening Design

The Plackett–Burman design matrix with responses obtained from the experimental study is given in Table 3. Entrapment efficiencies and particle size vary between 40.21 and 73.83% and 142 and 1363 nm, respectively.

**Table 3:** Entrapment Efficiency (%) and Particle Size (nm) of Sinapic-Acid-Loaded Nanoparticles Obtained with the Plackett–Burman Design

exp. no.	*X*_p1_	*X*_p2_	*X*_p3_	*X*_p4_	*X*_p5_	*X*_p6_	*X*_p7_	*R*1: entrapment efficiency (% EE)	*R*2: particle size (nm)
1	–1	–1	–1	–1	–1	–1	–1	40.21	161.9
2	1	–1	–1	1	–1	1	1	59.78	346.0
3	1	1	–1	–1	1	–1	1	55.04	212.0
4	1	1	1	–1	–1	1	–1	55.88	152.2
5	–1	1	1	1	–1	–1	1	73.83	619.5
6	1	–1	1	1	1	–1	–1	53.29	167.5
7	–1	1	–1	1	1	1	–1	47.59	141.6
8	–1	–1	1	–1	1	1	1	60.25	1363.0
9	0	0	0	0	0	0	0	53.00	164.1
10	0	0	0	0	0	0	0	56.36	146.2
11	0	0	0	0	0	0	0	60.21	148.4

#### 3.1.1 Development of the Regression Model and Statistical Analysis

Experimental data were transferred into Design Expert (13.0) software, and then the linear model for entrapment efficiency and particle size in terms of coded factors was developed, as shown in eq 6 and 7, respectively
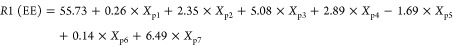
6
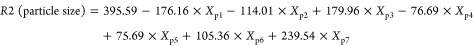
7The significance of the models was investigated with the analysis of variance (ANOVA) given in Table 4. *F* values of 7.44 and 1847.77, respectively, indicated that the developed models were significant and accurate with high regression coefficients. The developed model for the particle size had a 99% confidence level (*p*-value: 0.0005), whereas it had an 87% confidence level (*p*-value: 0.1235) for the developed model for EE. Hence, it was concluded that both models were statistically significant. Moreover, the determination coefficient (*R*^2^) of developed models was 0.9630 for EE% and 0.9996 for particle size, which indicated a good correlation between the observed and the predicted values.

**Table 4:** ANOVA Table of Developed Models Constructed by the Plackett–Burman Design

	model R1 (EE)	model R2 (size)
*R*^2^	0.9630	0.9998
standard deviation	3.61	9.76
*F*-value	7.44	1877.07
*P*-value	0.1235	0.0005

After implementing the screening design, the most effective three parameters were determined for further examination with response surface methodology. According to the obtained models, the mixing rate (*X*_p7_) and sonication power (*X*_p3_) were the most effective parameters in both models eqs 6 and 7. The next most effective parameters were sonication time (*X*_p4_) and the amount of PLGA (*X*_p1_) for the EE and particle size, respectively. To eliminate one of them, the relative magnitude of the coefficients was considered; hence, the amount of PLGA (*X*_p1_) was determined as the third parameter to be further investigated.

### 3.2 Box–Behnken Design

#### 3.2.1 Development of the Regression Model for Entrapment Efficiency and Size

In the Box–Behnken design, the effects of main parameters (amount of PLGA (X_b1_), mixing rate (X_b2_), and sonication power (X_b3_)) on entrapment efficiency and particle size were examined with 17 experiments, including a 5-center point experiment. In this design, experiments were carried out keeping other parameters constant (80 mg of sinapic acid, 1.5% of PVA, 60 s of sonication time, 2 mL/min flow rate 1 and 5 mL/min flow rate 2) at the level of the center points of the Plackett–Burman design, as shown in Table 1. All experimental data are reported in Table 5; as can be seen, entrapment efficiencies are between 45.79 and 61.88%, and sizes are between 149.5 and 170.8 nm, which are in the acceptable size range for drug delivery systems.

**Table 5:** Entrapment Efficiency and Particle Size (nm) of Sinapic-Acid-Loaded Nanoparticles Obtained with the Box–Behnken Design

exp. no.	*X*_b1_ (amount of PLGA)	*X*_b2_ (mixing rate)	*X*_b3_ (sonication power)	*R*1: EE (%)	*R*2: particle size (nm)
1	0	0	0	54.74	149.9
2	0	1	1	59.49	170.8
3	1	1	0	61.88	157.3
4	1	0	1	58.09	163.7
5	–1	–1	0	48.42	150.4
6	0	1	–1	57.88	164.4
7	0	0	0	51.36	152.3
8	–1	1	0	59.23	154.8
9	1	0	–1	48.78	157.9
10	–1	0	–1	55.58	149.5
11	–1	0	1	51.64	149.6
12	0	0	0	52.30	158.9
13	1	–1	0	53.68	161.3
14	0	–1	–1	45.79	145.0
15	0	0	0	52.19	159.3
16	0	–1	1	48.64	153.7
17	0	0	0	56.34	158.0

The developed quadratic model in terms of coded factors for entrapment efficiency and particle size are given in eqs 8 and 9, respectively. The reduced model developed for the particle size eq 9 was obtained by discarding statistically insignificant terms in the model.
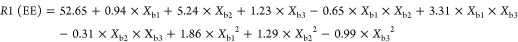
8
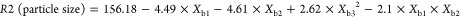
9

The significance of the models was investigated with ANOVA, as given in Table 6. *F* values of both models (12.19 and 3.83) demonstrated that the models were significant, and there were only 0.33 and 3.47% chances of error for the models of EE and particle size, respectively. Consequently, both developed models were statistically significant at a 95% confidence level.

**Table 6:** ANOVA Table of Models Constructed with the Box–Behnken Design

	model *R*1 (EE)	model *R*2 (size)
*R*^2^	0.9482	0.582
standard deviation	1.68	5.14
*F*-value	12.19	3.83
*P*-value	0.0033	0.0347

Moreover, the fitting of the models to the experimental data was examined with error analysis. As shown in Table 7, error values are between 0.51 and 14.58%, and the average error is 2.58% for entrapment efficiency. For the particle size, they are 0.1 and 5.0%, and the average error is 2.3%, which meant both developed models fitted experimental data very well.

**Table 7:** Error Analysis of Developed Models Constructed with the Box–Behnken Design

EE (%) (exp.)	EE (%) (predict.)	error (%)	size (nm) (exp.)	size (nm) (predict.)	error (%)
60.79	51.93	14.58	158.9	156.2	1.7
59.49	59.11	0.64	170.8	163.4	4.3
61.88	61.34	0.88	157.3	165.3	5.0
58.09	59.01	1.58	163.7	163.3	0.3
48.42	48.96	1.12	150.4	147.1	2.2
57.88	57.28	1.05	164.4	158.2	3.8
53.81	51.93	3.50	158	156.2	1.2
59.23	60.75	2.57	154.8	156.3	0.9
48.78	49.93	2.35	157.9	158.0	0.1
55.58	54.66	1.65	149.5	149.1	0.3
51.64	50.49	2.22	149.6	154.3	3.2
51.36	51.93	1.10	152.3	156.2	2.5
53.68	52.16	2.84	161.3	156.1	3.3
45.79	46.17	0.82	145	148.9	2.7
52.19	51.93	0.51	159.3	156.2	1.9
48.64	49.25	1.24	153.7	154.2	0.3
54.74	51.93	5.14	149.9	156.2	4.2

Considering the coefficient of parameters in eq 9, it can be concluded that the most effective parameter in entrapment efficiency is the mixing rate (*X*_b2_) with a coefficient of 5.24, while the least effective parameter is the amount of PLGA (*X*_b1_) with a coefficient of 0.95. However, the interaction effect of the PLGA amount (*X*_b1_) and sonication power (*X*_b3_) with a coefficient of 3.31 is higher than their individual linear and all other quadratic effects. In eq 9, the most effective parameter in the particle size is the mixing rate (*X*_b2_) with a coefficient of −4.61, while the least effective one is the sonication power (*X*_b3_) with a coefficient of 2.62. The PLGA amount with a coefficient of 4.49 is also very effective in particle size.

### 3.3 Response Surface Plots

**Figure 1:**
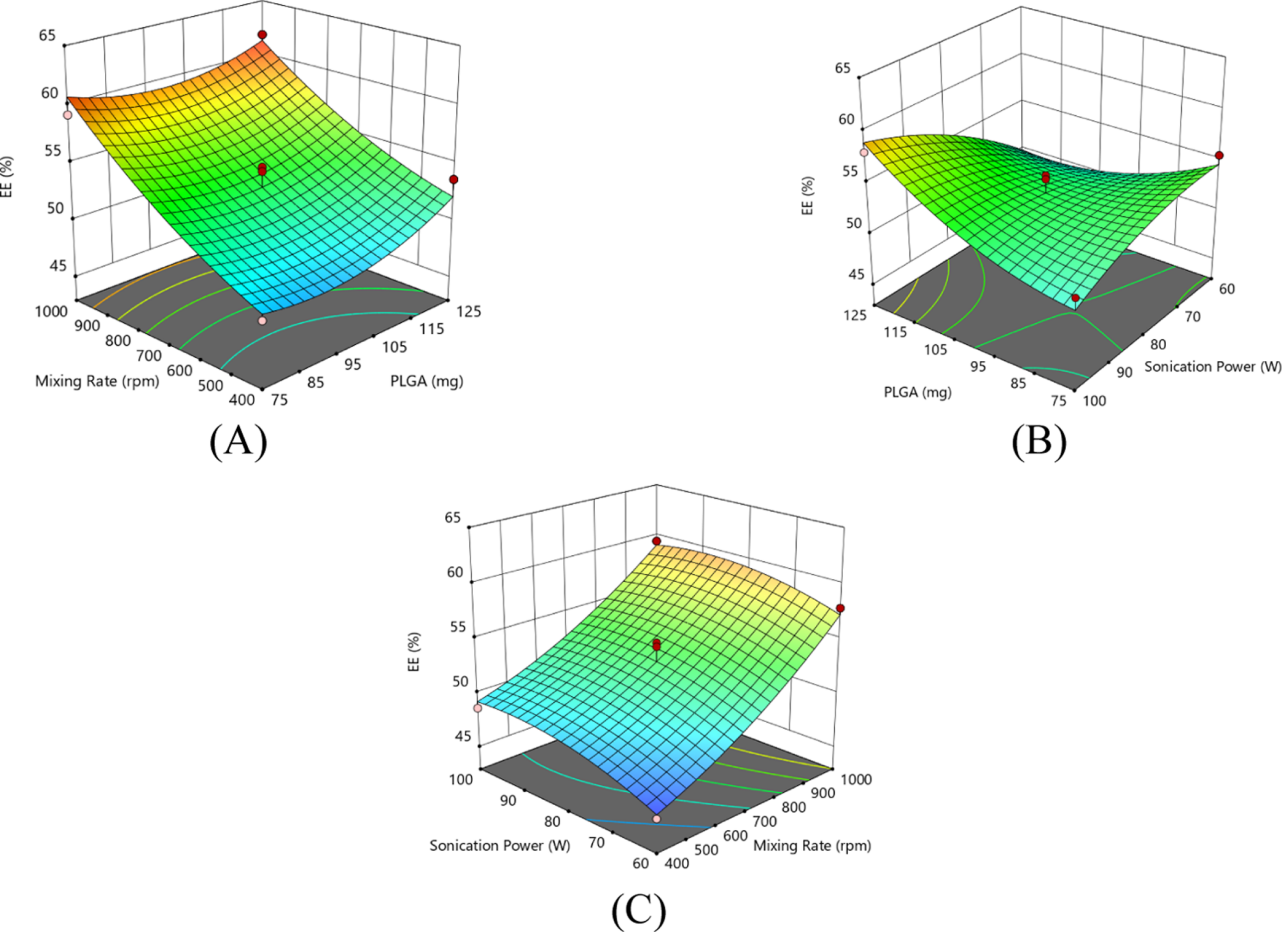
Response surface plots for entrapment efficiency as a function of process parameters: (A) mixing rate and PLGA amount, (B) amount of PLGA and sonication power, and (C) sonication power and mixing rate.

**Figure 2:**
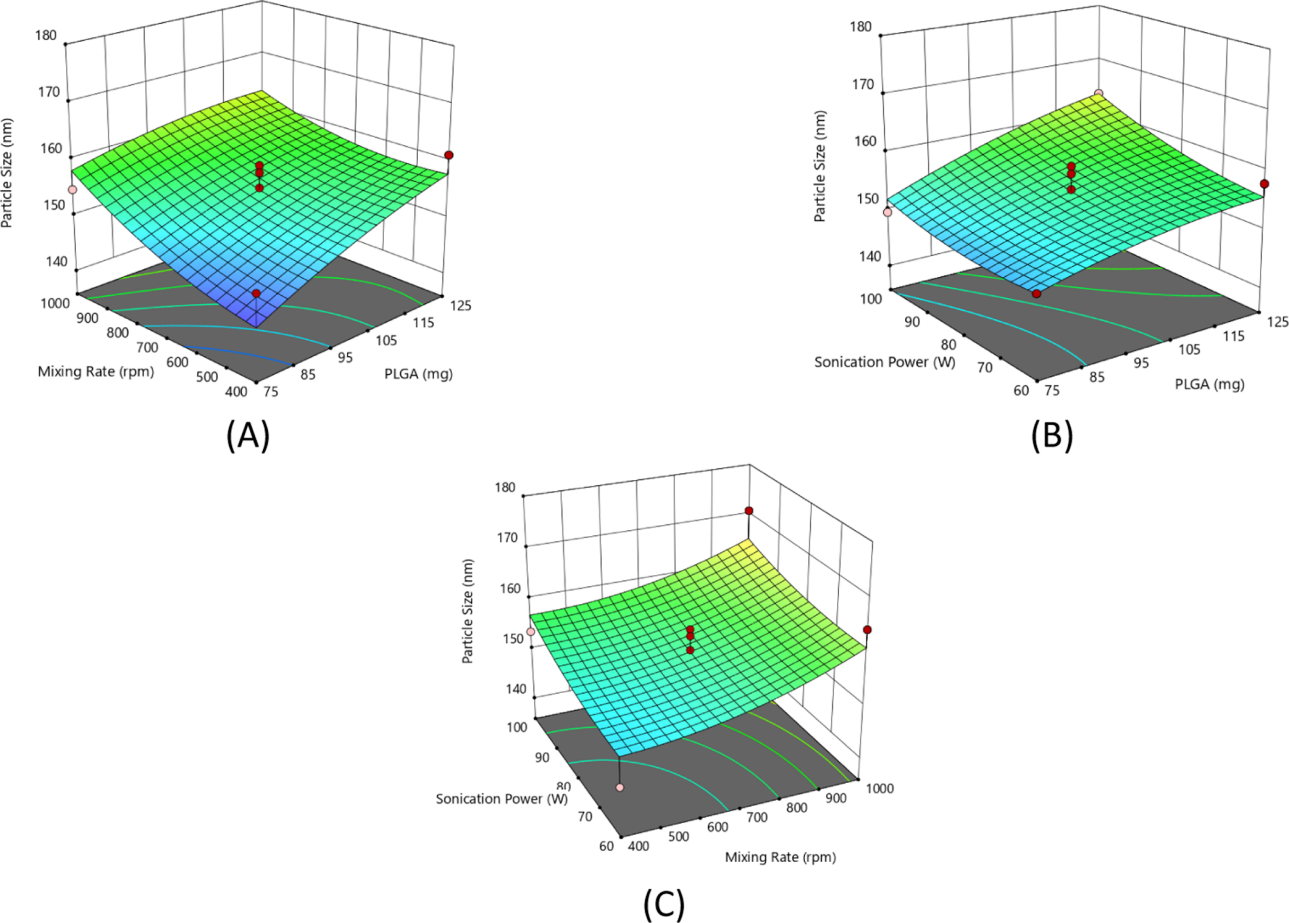
Response surface plots for particle size as a function of process parameters: (A) mixing rate and PLGA amount, (B) amount of PLGA and sonication power, and (C) sonication power and mixing rate.

The response surface method was used to evaluate the effects of three main parameters on EE and particle size. In order to identify the relation between the parameters and responses, regression surfaces were plotted as a function of process parameters using Design Expert 13.0 software. Therefore, the linear and quadratic interactions of the parameters on EE (Figure 1) and nanoparticle size (Figure 2) were clearly observed.

#### 3.3.1 Effects of Process Parameters on EE

When examining the response surface plot correlating the entrapment efficiency with the amount of PLGA and mixing rate (Figure 1A), it can be clearly seen that with increasing the mixing rate, a greater amount of sinapic acid is entrapped into the nanoparticle system. The amount of PLGA and its interaction with the mixing rate is only sligthly effective in EE.

The response surface plot (Figure 1B) correlating the EE with the amount of PLGA and sonication power shows that the EE value increases with increasing PLGA at high values of sonication power, whereas a high amount of PLGA causes a decrease in the EE value at low values of sonication power.

When the response surface plot given in Figure 1C is examined, it can be seen that the increasing mixing rate increases the EE value individually, but increasing sonication power is not significantly effective in EE.

#### 3.3.2 Effects of Process Parameters on Particle Size

When examining the response surface plot (Figure 2A) correlating the particle size with the mixing rate and the amount of PLGA, it can be clearly seen that PLGA and the mixing rate significantly affect the particle size; i.e., the smallest nanoparticle size can be obtained with the lowest value of both parameters.

As shown in Figure 2B, particle size significantly increases with increasing sonication power and PLGA amount, individually. Moreover, the binary interaction of these two parameters has a significantly increasing effect on the size eq 9.

Similar binary interaction effects on particle size can be concluded for all given response surface plots examined (Figure 2A–C). In addition, increasing sonication power and mixing rate resulted in an increase in particle size (Figure 2C).

### 3.4 Optimization of Process Parameters

Entrapment efficiency and nanoparticle size are two of the most important key points for drug delivery systems. Not only does obtaining high entrapment efficiency have advantages in the aspect of the economy, but also having reasonable particle size applicable for the drug delivery system is needed for the real application. After establishing the relationship between the main effects (amount of PLGA, mixing rate, sonication power) and both responses, we concluded that each process parameter had different effects on different responses; therefore, optimization was required in order to identify the optimum process condition. In order to obtain smaller nanoparticles with a higher amount of sinapic acid, both parameters were optimized by using Design Expert (13.0) software. A desirability function was used to find the levels of process parameters that produced the maximum entrapment efficiency and minimum particle size, spontaneously.

Sinapic-acid-loaded polymeric nanoparticles were synthesized in triplicate in optimal conditions to confirm the optimum point of the factors, and the results are given in Table 8. As can be seen from the table, the optimum parameters were obtained by using nanoparticles with an average size of 170 nm with an approximately 63% encapsulation efficiency with small errors such as 3.575–1.515%, respectively. The drug loading was 56% for the optimized nanoparticle.

**Table 8:** Validation of the Optimized Formulation

optimum parameters				predicted		experimental (average)		% error	
PLGA (mg)	sonication power (W)	mixing rate (RPM)	desirability function	EE (%)	size (nm)	EE (%)	size (nm)	EE (%)	size (nm)
116.73	96.72	995.62	1	62.0397	164.533	63.357 ± 2.12	170.633 ± 5.94	1.515	3.575

### 3.5 Characterization of Nanoparticles Synthesized at the Optimized Condition

**Figure 3:**
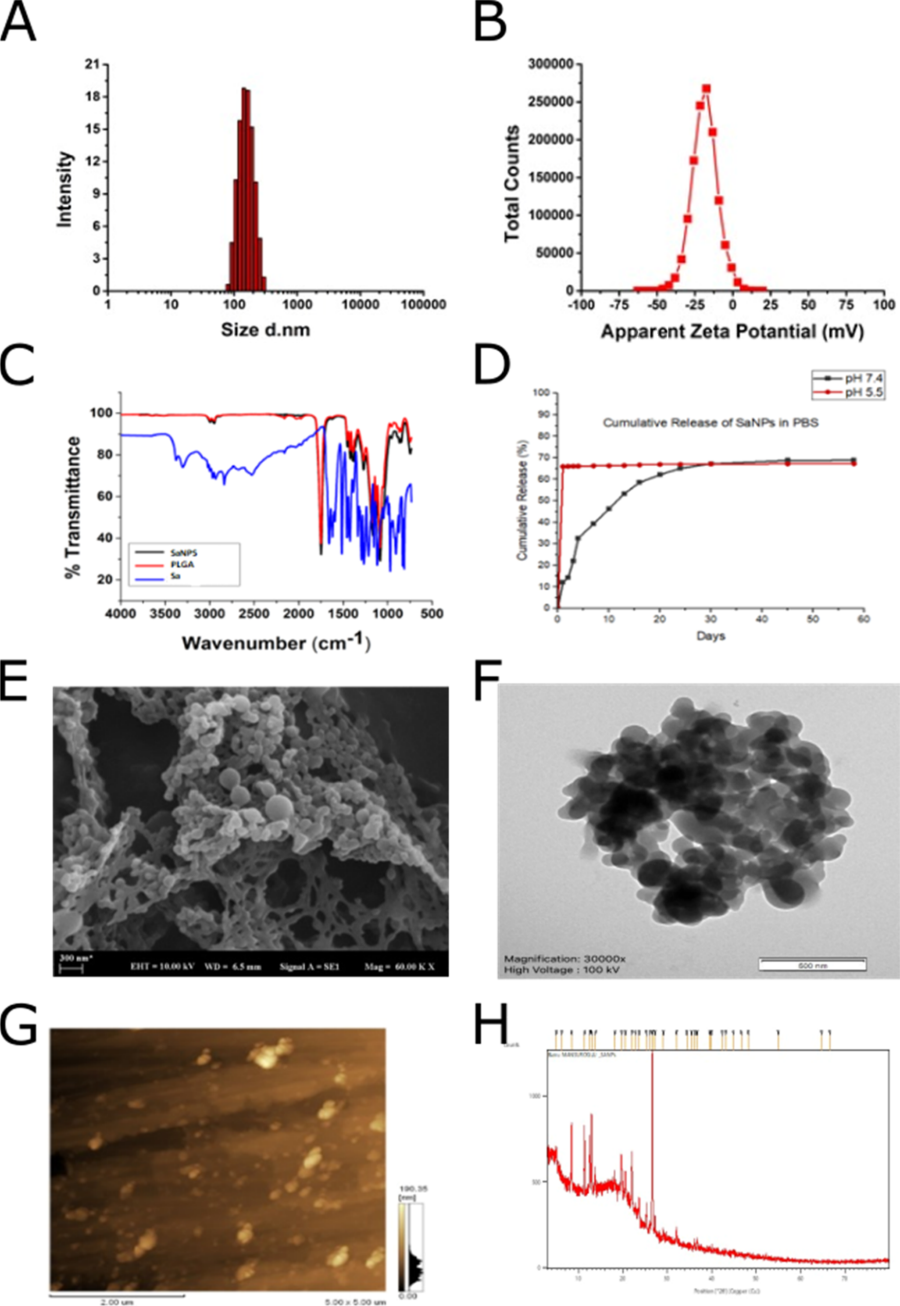
Characterization studies of optimized nanoparticles. (A) Particle size distribution of optimized nanoparticles. (B) ζ-potential of optimized nanoparticles. (C) FTIR spectra of sinapic acid, PLGA, and sinapic-acid-loaded nanoparticles. (D) Time-dependent release profile of nanoparticles in a PBS solution with pH 7.4 and pH 5.5. (E) SEM image of the optimized nanoparticles with a magnification rate of 60,000×. (F) TEM image of the optimized nanoparticles with a magnification rate of 30,000×. (G) AFM image of the optimized nanoparticles. (H) XRD spectra of the optimized nanoparticles.

Particle size distribution and ζ-potential measurement of the optimized nanoparticles were performed with a Zetasizer (Malvern ZEN 3600 Nano iS10). The average particle size of the optimized nanoparticles was measured as 170.633 ± 5.94 nm, the mean ζ-potential value was −17.37 ± 0.52, and the multiple distribution indices determining the size distribution were measured as 0.185. Particle size distributions and ζ-potentials of optimized nanoparticles are shown in Figure 3A,B, respectively.

FTIR spectra of the free sinapic acid molecule and PLGA and sinapic-acid-loaded optimized nanoparticles were drawn in order to determine the active substance–polymer interactions and to detect the active substance contained in/on the surface of the produced optimized nanoparticles. Figure 3C shows the FTIR spectrum graph of free sinapic acid, PLGA polymer, and sinapic-acid-loaded nanoparticles. As a result of the analysis of the obtained FTIR spectra, it was observed that the optimized nanoparticles had PLGA characteristics, and, therefore, the sinapic acid was effectively trapped in the nanoparticular system.

When the FTIR spectrum of the PLGA polymer was examined, it was seen that peaks of C–H stretching of C–H and CH_2_ were in the range of 2993–2989 cm^–1^, respectively. It was observed that the sharp and strong peak at around 1750 cm^–1^ was the peak of the C=O bond in the ester, and the peak in the range of 1160–1090 cm^–1^ was the tension band peak of the C–O bond.^28^ When the FTIR spectrum of the sinapic acid molecule was examined, it was thought that the peak at 3600 cm^–1^ was caused by −OH stress, and the wide and severe peaks in the 1600–1750 cm^–1^ range were caused by C=O, C=C, and C–O stresses. Similar peaks were also reported by Comunian in which Sa was used as a cross-linker in the encapsulation of various phenolic compounds into natural polymers.^29^ When the FTIR spectra were examined comparatively, the fact that the peaks of the characteristic stress and bending vibrations in the range 1600–1750 cm^–1^ were not observed in the FTIR spectrum of the nanoparticles showed that Sa was successfully loaded into the nanoparticles without surface adsorption (Figure 3C).

The release experiments of the optimized nanoparticles, which were suspended in PBS buffer (pH: 7.4 and pH: 5.5) and allowed to shake horizontally at 200 rpm at 37 °C, were performed for a period of 60 days. As shown in Figure 3D, at pH 7.4, approximately 69% of Sa was released gradually in a controlled manner within 30 days. On the other hand, it was observed that 44.8% of Sa was released within the first hour at pH 5.5, and a total of 67.2% of Sa within 24 h was released quickly.

The shape and surface morphology of optimized nanoparticles were investigated by using scanning electron microscopy (SEM), transmission electron microscopy (TEM), X-ray powder diffraction (XRD), and atomic force microscopy (AFM). Polymeric nanoparticles were made conductive after gold coating, and imaging was performed. Images of nanoparticles were taken on a Carl-Zeiss EVO LS10 scanning electron microscope with 10 kV and a magnification rate of 60,000× (Figure 3E) and a JEOL-JEM 1220 transmission electron microscope with 100 kV and a magnification rate of 30,000× (Figure 3F). When the SEM and TEM images of the optimized particles were examined, it was observed that the nanoparticles produced were in spherical morphology and homogeneous size distribution, and the size results were similar to those obtained by the dynamic light scattering method. In addition, it was found that the dimensions of the nanoparticles were compatible with the hydrodynamic size results obtained by the dynamic light scattering method by scaling the same SEM and TEM images.

The AFM image of the nanoparticles analyzed noncontact on the Shimadzu Scanning Probe Microscope-SPM 9600 atomic force microscope is given in Figure 3G. The XRD pattern of the SaNPs is demonstrated in Figure 3H.

### 3.6 Cell Culture Studies

#### 3.6.1 Cytotoxic Effect of Sa and SaNP on MCF-7 and MCF-10A Cells

**Figure 4:**
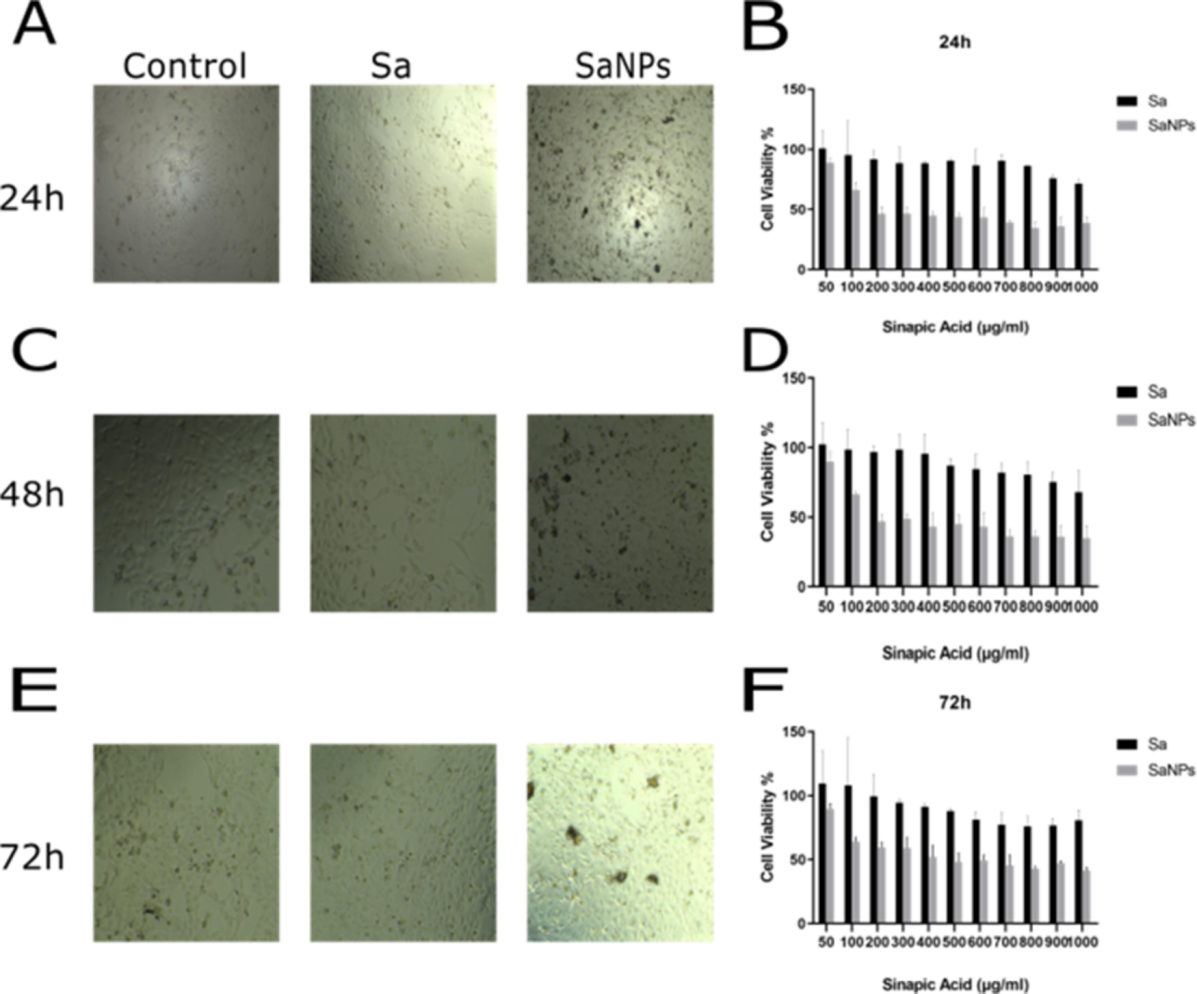
Representative images of cell viability at 24 h (A), 48 h (C), and 72 h (E). Cell viability at different concentrations (0–1000 μg/mL) of Sa and SaNP at 24 h (B), 48 h (D), and 72 h (F) applied to MCF-7 cells.

**Figure 5:**
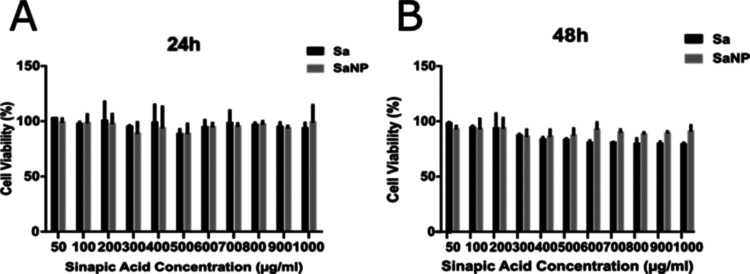
Cell viability graphs of Sa and optimized SaNP in MCF10A cells: (A) 24 and (B) 48 h.

The cytotoxic effect of doses of Sa and optimized SaNP formulations at different concentrations (0–1000 μg/mL) on human breast cancer MCF-7 (Figure 4) and nontumorigenic breast epithelial MCF10A (Figure 5) cell lines was determined by MTT assay.

At the 24th hour, free Sa started to inhibit MCF-7 cells at a concentration of 500 μg/mL and above, and at a concentration of 1000 μg/mL, it was observed that Sa inhibited 29% of MCF-7 cells. The IC_50_ value of SaNP was found to be 180 μg/mL.

At the 48th hour, the free Sa molecule appeared to have inhibited 32% of MCF-7 cells at a concentration of 1000 μg/mL. In MCF-7 cells, the IC_50_ value of SaNP was 168 μg/mL.

At 72 h, free Sa inhibited 47% of the MCF-7 cells at a concentration of 1000 μg/mL. The IC_50_ value of SaNP in MCF-7 cells was 145 μg/mL.

A study conducted by Raj et al. with Sa concentrations of up to 100 μg/mL in MCF-7 and MDA-MB 231 cells proved that it is nontoxic and recommended to study with higher Sa concentrations.^30^ There are similar studies in the literature where 8 different derivatives of sinapic acid are inhibited between 12 and 98% in a study on MCF-7 cells,^31^ and in another study, the Sa molecule was found to have IC_50_ values of 138.11 and 111.91 μg for the HT29 cell line and 112.55 and 98 μg for the SW-480 cell line at 24 and 48 h, respectively.^32^ In a study by which the anticancer mechanisms of sinapic acid were determined, the IC_50_ value of sinapic acid was found to be 1000 μM in PC-3 and LNCaP human prostate cancer cells by Eroğlu et al.^33^

When the potential colon and breast tumor suppressor properties of three different rice containing eight different phenolic compounds, including sinapic acid, were investigated, they weare found to reduce MDA-MB-468, HBL-100 breast cancer cells, and SW-480 colon cancer cells.^6^ Hameed et al. found that when the concentrations of sinapic acid were up to 500 and 2000 μM, it did not show a significant effect on V79 and HeLa cells, respectively, but IC_50_ values in V79 and HeLa cells at high concentrations were 1860 and 7248 μM, respectively.^1^

In a study similar to ours, Rahman et al. studied free sinapic acid and nanoencapsulated sinapic acid on Hep-2 cells and found IC_50_ values of 646.4 μM/mL for free sinapic acid and 84.74 μm/mL for the encapsulated form.^34^

As a result, SaNP containing the same concentration of Sa increased the cytotoxic activity of Sa on MCF-7 cells, significantly reducing cell viability over time compared with free Sa.

When the cell viability of the Sa molecule and SaNP at different concentrations (0–1000 μg/mL) was examined in MCF10A cells, no toxic effect was observed for 24, 48, and 72 h. Cell viability rates of MCF-10A treated with different concentrations of Sa and SaNP are given in Figure 5. The results for 48 and 72 h were found to be quite similar when compared.

Trimer and tetramer structures, which are different derivatives of the Sa molecule, were examined in cell proliferation in a study by Baltasa et al., indicating that Sa compounds are nontoxic in the MCF10A cell line.^35^ Similar to the data obtained in this study, there is research conducted by Reddy et al. that states that polyphenolic compounds that are beneficial for health do not have toxic effects on healthy cells, while they can have toxic effects on cancer cell lines.^36^

#### 3.6.2 Proliferation of Sa and SaNP on MCF-7 Cells

**Figure 6:**
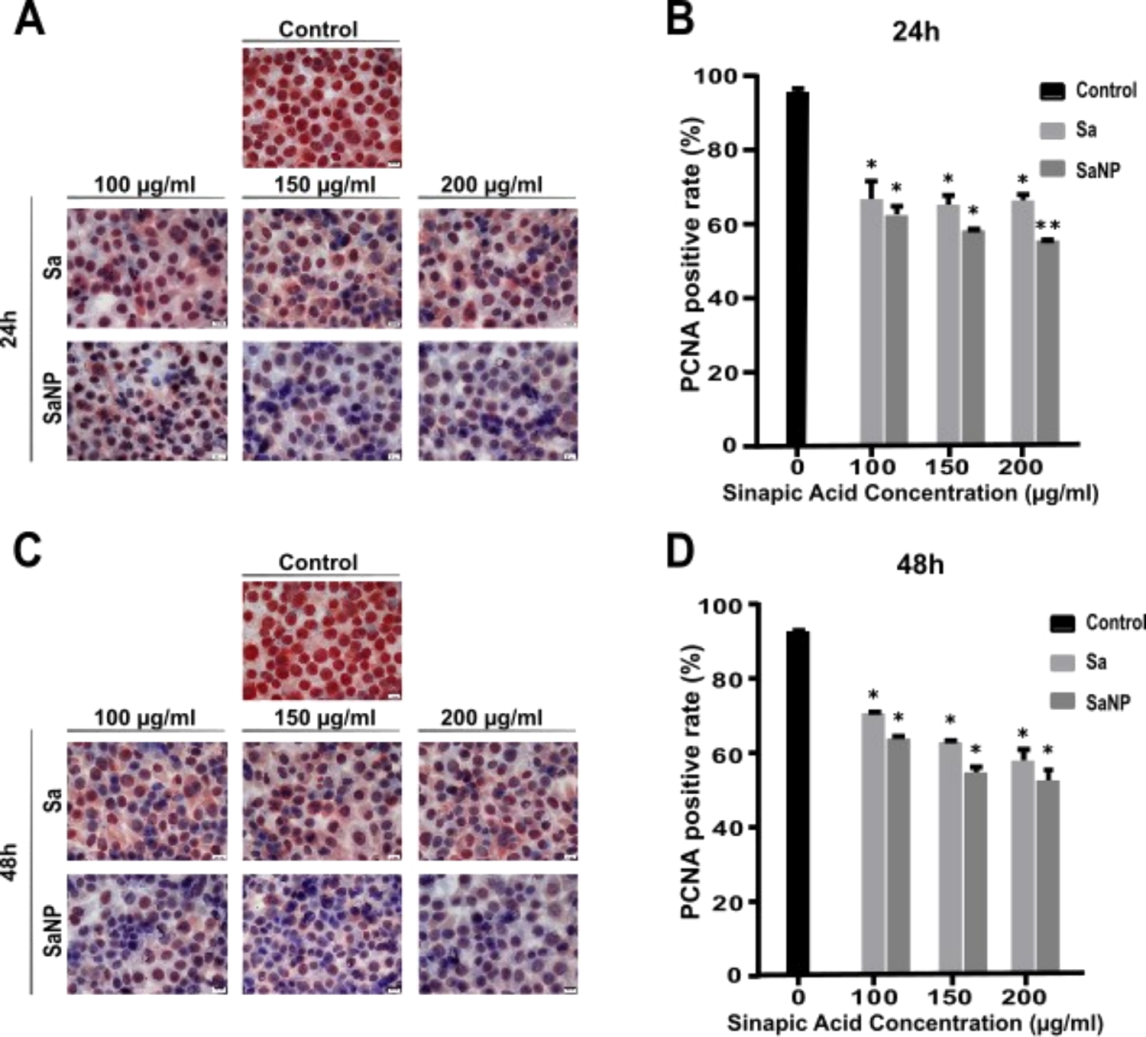
Immunocytochemical labeling of control MCF-7 cells treated with Sa and SaNP for 24 h (A) and 48 h (C). Proliferation of MCF-7 breast cancer cells treated with Sa and SaNP for 24 h (B) and 48 h (D).

The proliferating cell nuclear antigen (PCNA) was determined by the immunocytochemical method using a marker in therapy to specify the proliferation level of the cell after 24 and 48 h incubation of different concentrations of Sa and SaNPs (100–150–200 μg/mL) in MCF-7 cells (Figure 6A,C).

As a result of immunocytochemical labeling with PCNA, compared to control cells, a significant decrease in cell proliferation was detected in MCF-7 breast cancer cells treated with Sa and SaNP at all concentrations for 24 and 48 h (Figure 6B,D). As a result of treatment with Sa and SaNP, an administered concentration of 100–150–200 μg/mL was determined compared with control cells after 24 h of incubation where free Sa was 67.97, 64.43, and 66.27% and SaNP was 62.64, 58.56, and 55.37%, respectively.

Inhibition of cell proliferation was calculated as 70.33% at a concentration of 100 μg/mL, 62.21% at a concentration of 150 μg/mL, and 56.96% at a concentration of 200 μg/mL after 48 h of incubation, which was treated with free SA in MCF-7 human breast cancer cells compared to the control cells. SaNP was also determined as 63.29, 54.70, and 52%, respectively. Cell proliferation in MCF-7 human breast cancer cells treated with Sa and SaNP was significantly reduced when evaluated by the ANOVA test (*P < 0.05, **P < 0.03) (Figure 6). It has been found in studies that bioactive compounds such as quercetin, hesperetin, and caffeic acid phenyl ester (CAPE) and their nanoparticle formulations inhibit PCNA cell proliferation in various cancer cell lines such as breast (MCF-7), glioblastoma (C6), and colon (Caco-2).^16,37^

When the antiproliferative and apoptotic effects of selective phenolic compounds on T47D human breast cancer cells were investigated, it was shown that phenolics containing sinapic acid inhibited T47D cell proliferation in a dose-dependent and time-dependent manner.^38^

The efficiency of sinapic acid in pBR322 plasmid DNA in the presence of Cu + 2 was tested, and it was discovered to be much more effective in DNA damage than other phenolic compounds against human leukemia cell proliferation (HL-60). Cell proliferation increased with the addition of exogenous Cu + 2 ions and a low quantity of sinapic acid, but cell proliferation was inhibited by a high concentration of sinapic acid.^39^

In a study with *Hydnophytum formicarum* Jack. roots, whose main phenolic component is sinapic acid, antiproliferative effects were investigated in 5 different human cancer cells, that is, HeLa, HT29, HCT116, MCF-7, and Jurkat, and healthy Vero cells. *H. formicarum* Jack. roots prevented proliferation, especially in HeLa cells, and the antiproliferative effect was observed in other cancer cells; no toxic effect was observed in Vero cells.^40^

There are no studies that have been conducted in the literature showing the antiproliferative effect of Sa nanoparticles on MCF-7 cells. In this study, SaNP treatment caused a time-dependent decrease in proliferating cell numbers in MCF-7 cells due to Sa release.

#### 3.6.3 Apoptotic Effect of Sa and SaNP on MCF-7 Cells

**Figure 7:**
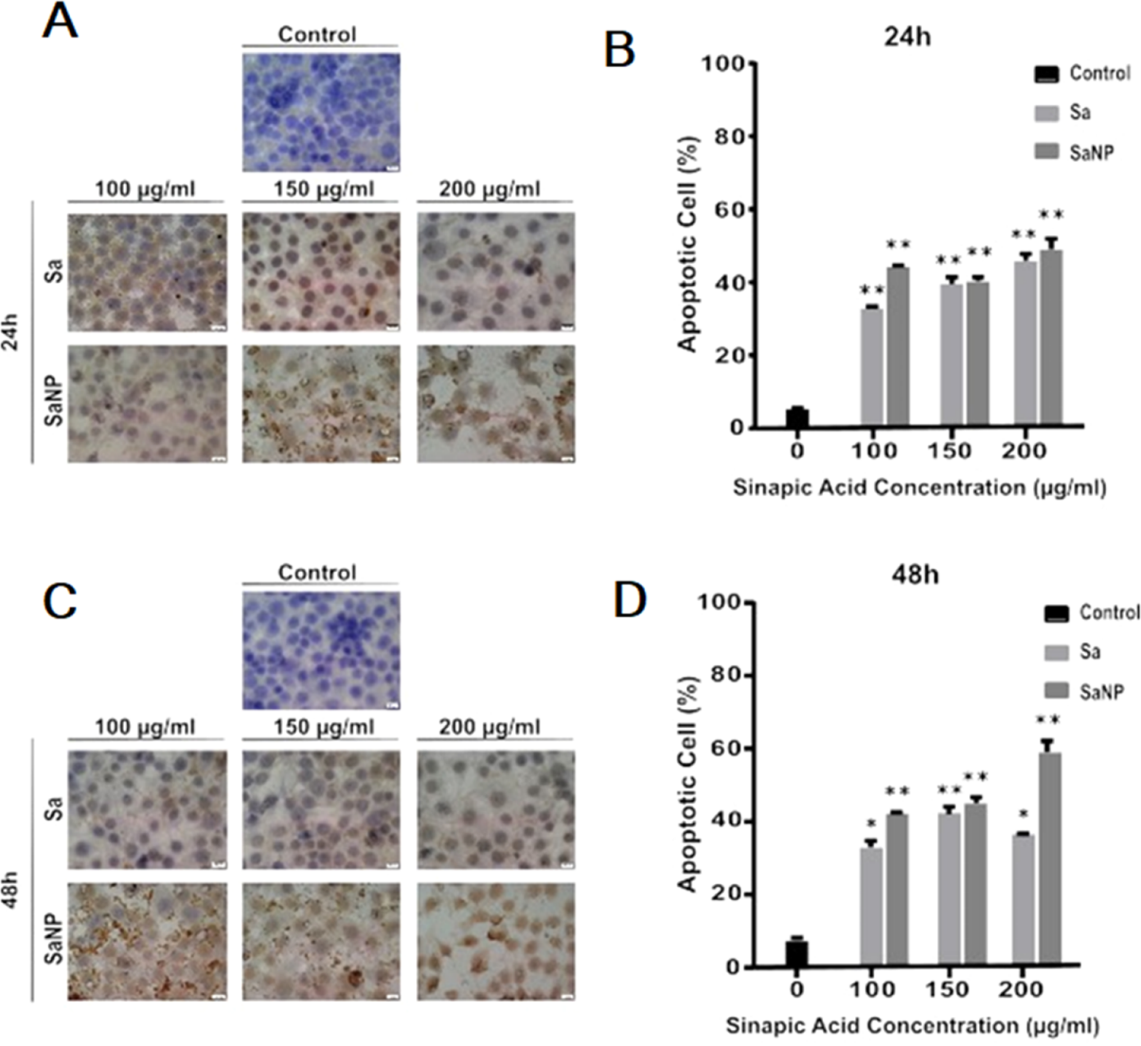
TUNEL (+) marking of MCF-7 cells treated with Sa and SaNP for 24 h (A) and graph for the apoptotic effect of MCF-7 breast cancer cells treated with Sa and SaNP for 24 h (B). TUNEL (+) marking of MCF-7 cells treated with Sa and SaNP for 48 h (C) and graph for the apoptotic effect of MCF-7 breast cancer cells treated with Sa and SaNP for and 48 h (D).

The apoptotic effect of different concentrations of Sa and SaNPs (100–150–200 μg/mL) in human breast cancer MCF-7 cells was demonstrated using the TUNEL method (Figure 7). The MCF-7 breast cancer cell line was treated with Sa and SaNP for 24 and 48 h, and the apoptotic cell number was determined.

Compared to the control cells at 24 h, apoptotic cells were calculated as 33.66, 37.28, and 46.42% for Sa and 44.83, 39.13, and 48.80% for SaNP, respectively. Similarly, for 48 h, apoptotic cell percentages were 33.69, 41.78, and 37.06% for Sa doses and 41.98, 42.93, and 58.72% for SaNP doses, respectively.

The ANOVA test showed that there was a statistically significant increase in the rate of apoptosis in MCF-7 breast cancer cells of Sa and SaNPs at concentrations of 100, 150, and 200 μg/mL treated for 24 and 48 h (*p < 0.05, **p < 0.03) (Figure 7B,D).

Kampa and Alexaki et al. reported that some phenolic compounds, which have a sinapic acid, have been known to show antiproliferative and apoptotic effects on T47D human breast cancer cells at different concentrations and incubation times.^38^ In another study, similarly, they have been reported to modulate the signal pathways of the cell, activate cell death signals, and initiate apoptosis in preneoplastic or neoplastic cells, thereby inhibiting cancer development and progression.^41^ When the renoprotective functions of sinapic acid and the pathways mediating this function were examined to evaluate the nephrotoxicity caused by gentamicin, it was seen that it reduces kidney failure and structural injuries through oxidative/nitrosative stress, inflammation, and downregulation of apoptosis.^42^ Despite all of these studies, there is no study in the literature indicating directly that sinapic-acid-loaded nanoparticle systems induce apoptosis.

The results of this study show that Sa and SaNPs effectively induce apoptosis in MCF-7 cancer cells. Cell proliferation decreased depending on the number of cells undergoing apoptosis.

#### 3.6.4 Biochemical and Antioxidant Activities of Sa and SaNP

Oxidative stress contributes to the pathophysiology of several illnesses, including aging, cancer, atherosclerosis, diabetes, neurological, liver, and renal disorders. Dietary antioxidants that protect against oxidative stress-related disorders are organic compounds that shield body cells from free radicals and reactive oxygen species (ROS).^43^

Thanks to the hydroxyl group in the structure of the sinapic acid molecule, which has a monophenolic structure, it can exchange electrons and have an oxidizing/reducing effect. Due to this feature, the molecule shows an antioxidant effect.^44^

Biochemical and antioxidant parameters were measured in cell lysates obtained from MCF-7 cells treated with Sa and SaNP at concentrations of 100, 150, and 200 μg/mL for 24 and 48 h.

##### 3.6.4.1 Measurements of Malondialdehyde (MDA) Levels

In vitro anticancer studies have shown that increased ROS levels and lipid peroxidation byproducts found in primary cancer cells are associated with a reduction in antioxidants such as SOD and CAT. In lipid peroxidation, a free-radical-mediated process, it is known that biological membranes deteriorate as a result of increased intracellular ROS levels, and this causes cytotoxicity.^45^

**Figure 8:**
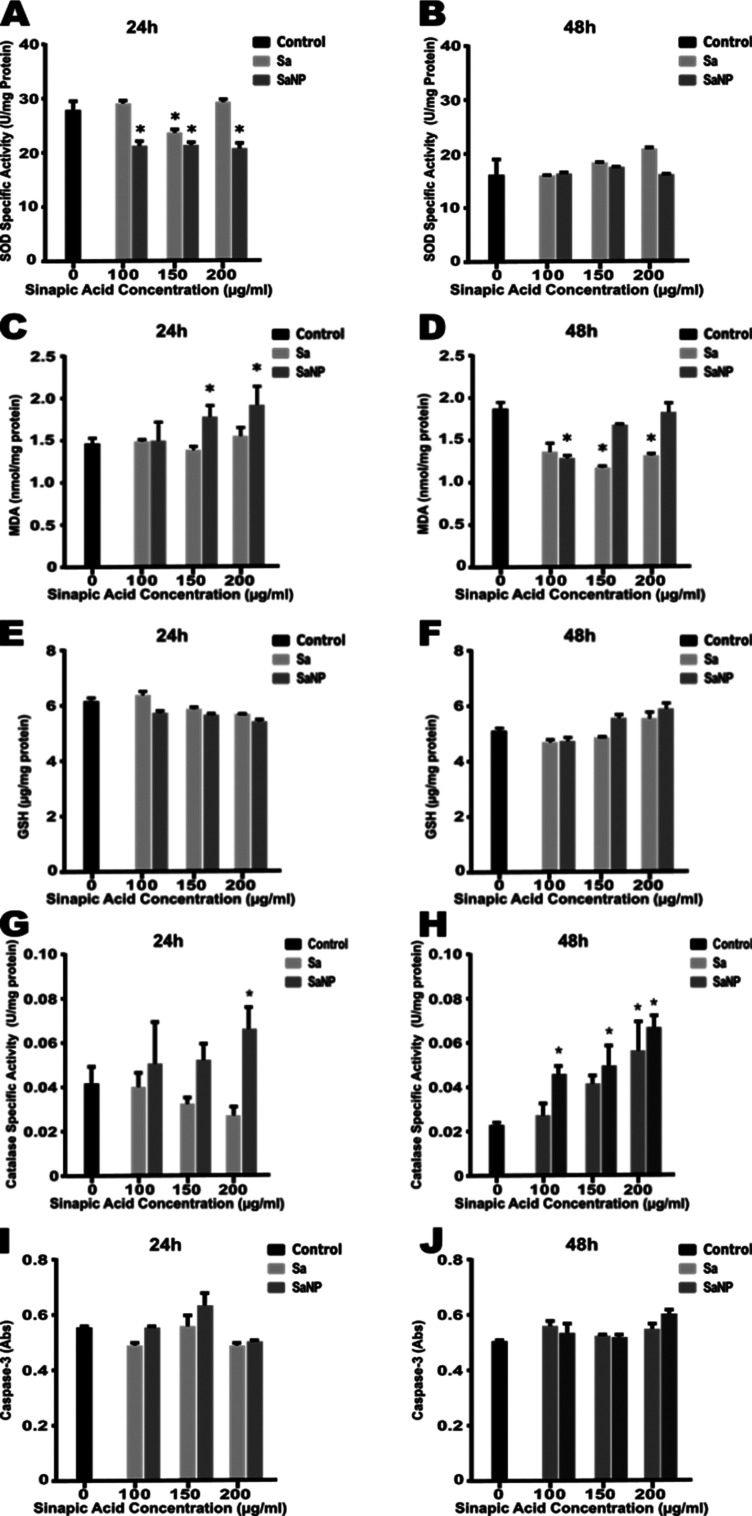
Biochemical and antioxidant activities of optimized nanoparticles. (A, B) SOD activity of Sa and SaNP in MCF-7 cells. (C–D) MDA levels of Sa and SaNP in MCF-7 cells. (E, F) GSH levels of Sa and SaNP in MCF-7 cells. (G, H) Catalase activities of Sa and SaNP in MCF-7 cells. (I, J) Caspase-3 activities of Sa and SaNPs in the MCF-7 cell (all graphs are given for 24 and 48 h, respectively).

As a result of the ANOVA test, MDA levels, which are indicative of lipid peroxidation, were significantly increased in MCF-7 cells at 150 and 200 μg/mL concentrations of SaNPs treated for 24 h compared to control cells (*p* < 0.05). After 48 h of treatment at Sa 150 and 200 μg/mL and SaNP at 100 μg/mL, there was a significant decrease in MDA levels in MCF-7 cells compared to control cells (*p* < 0.05) (Figure 8C,D). According to our results, SaNP can be thought to direct the cell to apoptosis through ROS production.

##### 3.6.4.2 Superoxide Dismutase (SOD) Enzyme Activity

In vitro studies have shown that there is a decrease in the level of antioxidant enzymes such as SOD and catalase in response to the increased ROS level in cancer cells.^32^ SOD enzyme activity in MCF-7 cells at all concentrations of Sa and SaNP at 150 μg/mL for 24 h significantly decreased compared to the control (*p* < 0.05) (Figure 8A,B). Franco-Molina, et al. reported that MCF-7 cells examined the anticancer activity with colloidal silver. It was stated that SOD activity was increased, and other enzymatic antioxidants like GSH (Figure 8E,F) showed results similar to our study.^46^ This situation provided by GSH was thought to cause redox imbalance and increased H_2_O_2_ amount; therefore, it was stated that it directed cancer cells to apoptosis. However, there was no significant change in SOD activity, an antioxidant enzyme, compared to control in cells treated with SaNP at the same doses after 48 h of incubation.

##### 3.6.4.3 Determination of Glutathione (GSH) Levels

It is reported in the literature that the increase in the ROS level in cancer cells causes a decrease in the intracellular GSH level.^32^ GSH levels in MCF-7 cells were not statistically significant for Sa and SaNPs at all concentrations compared to the control (Figure 8E,F). While the level of GSH, which is a reducing agent that develops in response to oxidative stress, observed no significant decrease at different doses of Sa and SaNP in the MCF-7 cell line compared to the control group in 24 h, but then it was detected to increase in 48 h.

##### 3.6.4.4 Catalase Activity

In a study where antioxidant and oxidant efficacy was investigated in MCF-7 cells, it was stated that radical superoxides converted into H_2_O_2_ by SOD could be converted into water with the CAT enzyme, and in cases where low CAT activity was observed, they turned into hydroxyl radicals and caused the lipid peroxidation process.^47^ A significant increase in catalase enzyme activity was observed in MCF-7 cells at a 200 μg/mL concentration of SaNPs for 24 h compared to control cells (*p* < 0.05), (Figure 8G).

A significant increase in catalase enzyme activity was observed in MCF-7 cells at a 200 μg/mL Sa concentration and all SaNP concentrations for 48 h compared to control cells (*p* < 0.05), (Figure 8H).

Consequently, increased CAT enzyme activity is consistent with 48 h MDA results.

##### 3.6.4.5 Caspase-3 Activity

A study on the apoptosis mechanism of taxol, a chemotherapeutic agent, was found to be independent of caspase-3. A similar situation exists in MCF-7 cells studied with the designed PAMAM–drug–trastuzumab conjugates.^48,49^

Compared to the control cells, there was not a change in caspase-3 activity in MCF-7 cells at all concentrations of SA and SANP (Figure 8I,J).

In a study, assuming that the sinapic acid and resveratrol treatment may be a potential enhancer in the antioxidant effect and imbalance of the intestinal microbiota, a combination of resveratrol, sinapic acid, and both polyphenols was given together, while the fasting blood glucose levels in rats decreased effectively, while the reactive oxygen types and malondialdehyde levels decreased. It was observed that total antioxidant capacity in the liver increased with consumption of sinapic acid.^50^

In Pekkarinen et al.’s study, the ability of phenolic acids containing different concentrations of sinapic acid to inhibit lipid oxidation in homogeneous and heterogeneous lipid systems was evaluated, and sinapic acid was more effective than ferulic acid and α-tocopherol in both systems.^51^

Highly reactive hydroxyl radicals (^•^OH) have the potential to harm their environment in living systems. Compared to ascorbic acid, sinapic acid had a stronger antioxidant effect at lower doses for the ^•^OH radical.^52,53^

## 4 Conclusions

In this study, a sinapic acid molecule with broad-spectrum pharmacological properties was loaded into nanoparticular carrier systems by using experimental design methods in order to increase the bioavailability and biocompatibility and to increase stability in a biological system. Process parameters that provide maximum entrapment efficiency and minimum particle size for SaNP preparation were optimized by applying two successive experimental design methods. Cytotoxic effects were investigated by the MTT method of free Sa and optimized SaNPs on MCF-7 human breast cancer and MCF10A nontumorigenic breast epithelial cell lines. The antiproliferative effect was investigated with PCNA by the immunohistochemical method. Apoptotic cell death was investigated with TUNEL by the in situ method. Furthermore, the protective/restorative effect of Sa and SaNPs against oxidative damage in MCF-7 breast cancer cells was analyzed biochemically. SOD activity, catalase activity, GSH, MDA, and caspase-3 levels, which are indicative of apoptosis, were examined in these analyses.

It has been previously reported in the literature that nanoparticles smaller than 500 nm in size could be removed by cell endocytosis^54^ Also, with their small size, nanoparticles that can interact with cells in the tumor play an important role in passively targeting the tumor with an increased permeability and retention (EPR) effect.^55^ In recent years, studies with nanoparticle systems have increased considerably, and their positive effects are available in the literature. For example, PLGA-encapsulated gold quercetin nanoparticles have been shown to preferably kill HepG2 cells and reduce cell proliferation.^56^ Dikmen et al. determined the effects of caffeic-acid-loaded solid lipid nanoparticles on MCF-7 cells with the MTT test and Annexin V–PI analysis; as a result, it was determined that caffeic-acid-loaded nanoparticles were more effective in MCF-7 cells than free caffeic acid.^57^ In a study examining the effects of ferric-acid-loaded polymeric nanoparticles on hypoglycemia and wound healing, nanoparticles were administered orally and topically to rats, and the results showed that ferulic-acid-loaded nanoparticles significantly improved wound healing in diabetic rats.^58^ Ersoz et al. evaluated the anticancer properties of hesperetin and hesperetin-loaded PLGA nanoparticles. In their study, the nanoparticular system showed great advantages in increasing the anticancer activity of hesperetin and poor solubility of the therapeutic agent in glioblastoma cells.^16^

In this study, optimized nanoparticles produced using the experimental design methods have been shown to exhibit higher antiproliferative and apoptotic effects at lower doses in MCF-7 breast cancer cells than in the free molecule. In this study, it was observed that SaNP inhibited proliferation by 55.37% in 24 h and by 52% in 48 h. In the TUNEL method, in which apoptosis was determined, after 24 and 48 h of incubation, it was observed that the lowest dose of SaNP increased apoptosis approximately 5 and 6 times, respectively. Furthermore, SaNPs showed high antioxidant activity in MCF-7 cells, contributing to the elimination of free radicals and preventing the proliferation of cancer cells. Increased ROS activity enables the activation of pathways leading to apoptosis. In this study, the MDA level is compatible with TUNEL test results. Also, a decrease in the level of SOD and CAT contributes to the increase of the free-radical level and the orientation of the cell to apoptosis. In addition, the GSH level maintains the redox status of the cell. Apoptosis is thought to not use the caspase-3 pathway. It is important that these optimized sinapic-acid-loaded nanoparticles do not have a toxic effect on MCF-10A nontumorigenic mammary epithelial cell lineage and have an inhibitory effect on MCF-7 breast cancer cells.

Present research highlights the need to encapsulate sinapic acid in biocompatible nanoparticle systems in order to increase the molecule’s poor solubility, improve its availability in biological systems, and spread potential chemotherapeutic uses. The findings of this study may help in the formation of polyphenolic chemicals with nanoparticles and therapeutic designs for therapy. Furthermore, it is proposed that these research results can be enriched with subsequent in vivo experiments.

## Abbreviations

PLGA: poly(lactic-*co*-glycolic acid)
Sa: Sinapic acid
SaNPs: Sinapic-acid-loaded nanoparticles
EE: entrapment efficiency
DL: drug loading
PCNA: proliferating cell nuclear antigen
TUNEL: transferase-mediated dUTP end labeling
SOD: superoxide dismutase
MDA: malondialdehyde
GSH: reduced glutathione
